# Innovative preconditioning strategies for improving the therapeutic efficacy of extracellular vesicles derived from mesenchymal stem cells in gastrointestinal diseases

**DOI:** 10.1007/s10787-023-01350-6

**Published:** 2023-10-24

**Authors:** Manar A. Didamoony, Ayman A. Soubh, Ahmed M. Atwa, Lamiaa A. Ahmed

**Affiliations:** 1 Faculty of Pharmacy, Pharmacology and Toxicology Department, Egyptian Russian University, Cairo, 11829 Egypt; 2 Faculty of Pharmacy, Pharmacology and Toxicology Department, Ahram Canadian University, 4th Industrial Zone, Banks Complex, 6th of October City, Giza, 12451 Egypt; 3 Faculty of Pharmacy, Pharmacology and Toxicology Department, Cairo University, Cairo, 11562 Egypt

**Keywords:** Biological preconditioning, Extracellular vesicles, Gastrointestinal diseases, Mesenchymal stem cells, Pharmacological preconditioning

## Abstract

Gastrointestinal (GI) diseases have become a global health issue and an economic burden due to their wide distribution, late prognosis, and the inefficacy of recent available medications. Therefore, it is crucial to search for new strategies for their management. In the recent decades, mesenchymal stem cells (MSCs) therapy has attracted attention as a viable option for treating a myriad of GI disorders such as hepatic fibrosis (HF), ulcerative colitis (UC), acute liver injury (ALI), and non-alcoholic fatty liver disease (NAFLD) due to their regenerative and paracrine properties. Importantly, recent studies have shown that MSC-derived extracellular vesicles (MSC-EVs) are responsible for most of the therapeutic effects of MSCs. In addition, EVs have revealed several benefits over their parent MSCs, such as being less immunogenic, having a lower risk of tumour formation, being able to cross biological barriers, and being easier to store. MSC-EVs exhibited regenerative, anti-oxidant, anti-inflammatory, anti-apoptotic, and anti-fibrotic effects in different experimental models of GI diseases. However, a key issue with their clinical application is the maintenance of their stability and efficacy following in vivo transplantation. Preconditioning of MSC-EVs or their parent cells is one of the novel methods used to improve their effectiveness and stability. Herein, we discuss the application of MSC-EVs in several GI disorders taking into account their mechanism of action. We also summarise the challenges and restrictions that need to be overcome to promote their clinical application in the treatment of various GI diseases as well as the recent developments to improve their effectiveness.

**Graphical abstract:**

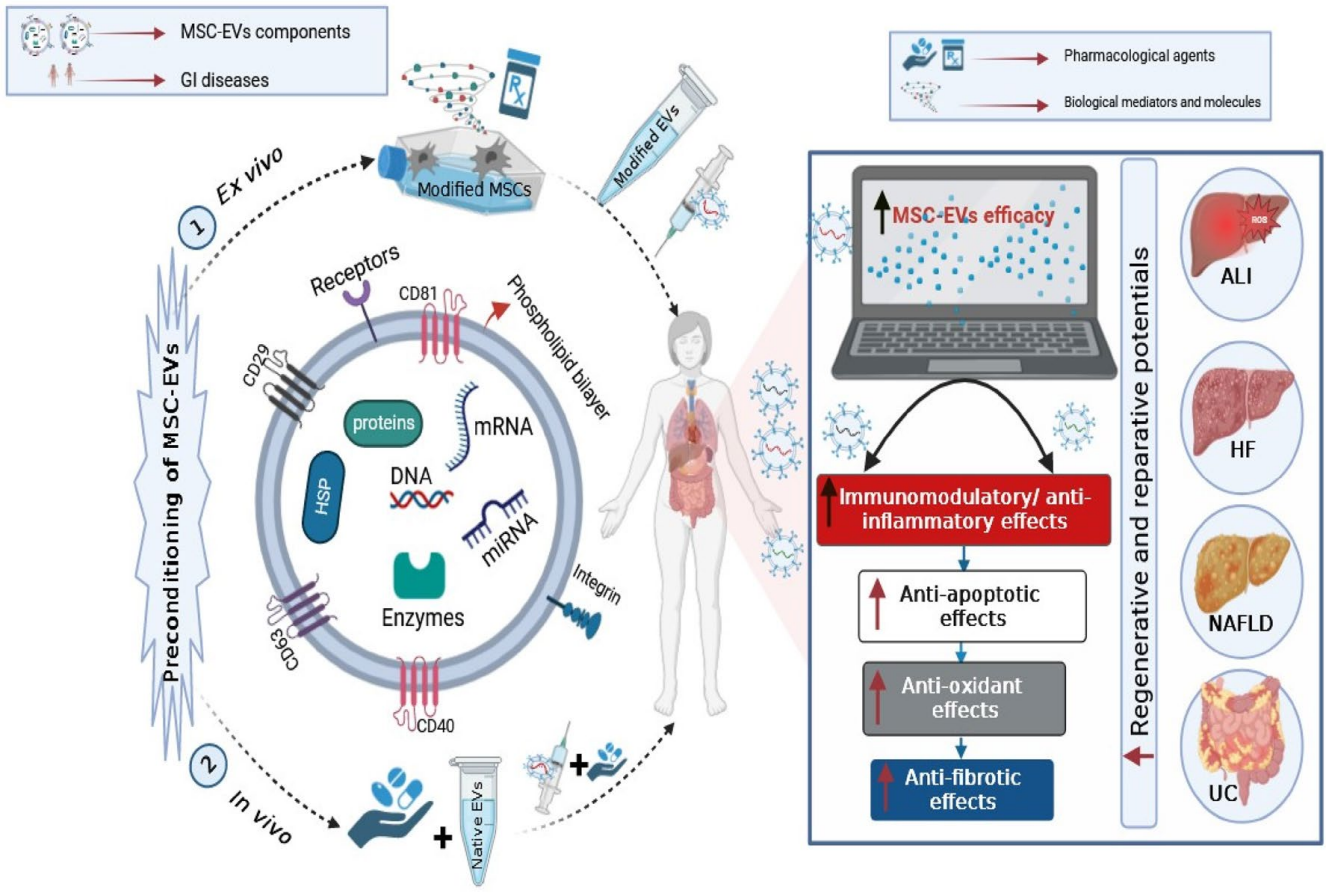

A representation of the innovative preconditioning techniques that have been suggested for improving the therapeutic efficacy of MSC-EVs in GI diseases. The pathological conditions in various GI disorders (ALI, UC, HF and NAFLD) create a harsh environment for EVs and their parents, increasing the risk of apoptosis and senescence of MSCs and thereby diminishing MSC-EVs yield and restricting their large-scale applications. Preconditioning with pharmacological agents or biological mediators can improve the therapeutic efficacy of MSC-EVs through their adaption to the lethal environment to which they are subjected. This can result in establishment of a more conducive environment and activation of numerous vital trajectories that act to improve the immunomodulatory, reparative and regenerative activities of the derived EVs, as a part of MSCs paracrine system. ALI, acute liver injury; GI diseases, gastrointestinal diseases; HF, hepatic fibrosis; HSP, heat shock protein; miRNA, microRNA; mRNA, messenger RNA; MSC-EVs, mesenchymal stem cell-derived extracellular vesicles; NAFLD, non-alcoholic fatty liver disease; UC, ulcerative colitis.

## Introduction

Gastrointestinal (GI) diseases are a series of inflammatory conditions which affect any section of the GI tract, from the oesophagus to the rectum, in addition to the accessory digestive organs—liver, gall bladder and pancreas. Motility problems, visceral hypersensitivity, altered mucosal and immunological function, and altered intestinal microbiota are all hallmarks of these conditions (Oshima and Miwa 2015; Drossman 2016). Irritable bowel diseases (IBD), gastroesophageal reflux disease, liver diseases, peptic ulcer, pancreatitis, and GI malignancy are just a few of many problems that fall under the umbrella of GI disorders, which affect patients worldwide (De Filippis et al. 2020). Many of these diseases negatively impact patients’ quality of life and productivity (Wang et al. 2023). Moreover, their incidence is high, and oftentimes, there are no obvious symptoms in the early stages; hence, most GI diseases are first noted in the middle and late stages where the prognosis is poor (Chen et al. 2022a) and are not effectively managed using current medications (Greenwood-Van Meerveld et al. 2017). As a result, it is crucial to create new and efficient strategies for treating GI disorders.

Over the past few decades, stem cell therapy has attracted attention as a viable option for treating a wide range of pathological conditions. Mesenchymal stem cells (MSCs) have been of particular importance because of their ability to self-renew and differentiate into a wide variety of cell types (Kou et al. 2022). They are commonly extracted from bone marrow (BM), amniotic fluid (AM), adipose tissues (AD), dental pulp, and umbilical cord (Wagner et al. 2005; Hass et al. 2011; Musiał-Wysocka et al. 2019). Notably, numerous preclinical and clinical studies have proved the potential role of MSCs in GI protection and repair (Kubo et al. 2015; Onishi et al. 2015; Ono et al. 2015; Trounson and McDonald 2015). It was once thought that MSCs’ therapeutic efficacy arises from their ability to migrate to and engraft in the target tissues. However, it was shown afterwards that the biological effects observed following MSCs administration are likely the result of their soluble secreted factors such as cytokines, chemokines, growth factors, and extracellular vesicles (EVs) (Keating 2012; Aghajani Nargesi et al. 2017; Gowen et al. 2020). These biological factors act either on MSCs themselves (autocrine functions) to maintain self-renewal capacity, differentiation, and proliferation or on neighbouring cells (the predominant paracrine functions) to modulate the immune system, inflammatory response, and apoptosis and to stimulate neo-angiogenesis (Razavi et al. 2020; Rahimi et al. 2021). Besides, EVs are the main component of paracrine actions of MSCs (Han et al. 2016).

EVs are membrane-bound nanovesicles (with a size range of 30–1000 nm) that transport vital biomolecules such as cytokines, growth factors, signalling lipids, messenger RNAs (mRNA), and micro-RNAs (miRs) between cells and regulate a wide range of cellular processes under both normal as well as pathological circumstances (Gowen et al. 2020; Heydari et al. 2021; Ahmed and Al-Massri 2022). MSC-derived EVs (MSC-EVs) are mainly made up of exosomes (EXOs), microvesicles (MVs), and apoptotic bodies (ABs). It is worth noting that MSC-EVs revealed regenerative, anti-oxidant, anti-inflammatory, anti-apoptotic, and anti-fibrotic effects in different experimental models of GI diseases such as IBD, severe acute and chronic pancreatitis, hepatic fibrosis (HF), acute liver injury (ALI), and non-alcoholic fatty liver disease (NAFLD) (Zhou et al. 2013; Yang et al. 2015; Mao et al. 2017; Xie et al. 2019a; Dong et al. 2020; Ren et al. 2021; Wu et al. 2021; Niu et al. 2022).

Increasing evidence suggested that MSC-EVs, rather than MSCs themselves, are responsible for the majority of the therapeutic actions of MSCs (Zhao et al. 2020a). Therefore, MSC-EVs can be used since they have a better safety profile, are less immunogenic, and can traverse biological barriers (Yeo et al. 2013; Natasha et al. 2014). In addition, problems of MSCs such as risk of ectopic tumour growth, entrapment in lung microvasculature, and immunological rejection could be avoided when MSC-EVs are used (Badillo et al. 2007; Jeong et al. 2011; Wang et al. 2015; Fennema et al. 2018). Despite these obvious potentials, the clinical application of MSC-EVs faces a number of obstacles, such as the inability of EVs to retain their efficacy and stability over time following in vivo transplantation (Wiklander et al. 2015). Therefore, new strategies, like preconditioning of MSC-EVs or their parent cells, could be explored to improve their effectiveness and stability upon application (Lee and Kang 2020).

In this review, we first summarised different approaches used for the isolation, characterisation, purification, and storage of MSC-EVs. We also illustrated their applications in different models of GI diseases and the underlying mechanisms of their bioefficacy. Besides, we discussed recent studies and methods aiming at the improvement of the therapeutic efficacy of MSC-EVs using different biological or pharmacological preconditioning approaches. Finally, we enumerated challenges and restrictions that should be overcome to promote the clinical application of MSC-EVs in various GI diseases.

## Biological properties of EVs

### Definition and origin of EVs

EVs are heterogenous nanoparticles circumscribed by a phospholipid membrane carrying transmembrane proteins, cytosolic proteins, organelles, transcription factors, mRNAs, miRs and various signal transduction molecules and are generally detected in MSCs, tumour cells, fibroblasts, neurons, endothelial cells, and epithelial cells, serving as versatile messengers between adjacent or distant cells in numerous pathological and physiological processes (Raposo and Stoorvogel 2013; Kalluri 2016; Keshtkar et al. 2018; Kou et al. 2022). Initially, EVs were noticed in the reticulocytes of sheep in the 1980s (Raposo and Stoorvogel 2013) and were thought to be secreted in order to eliminate unwanted compounds from the cell (Johnstone et al. 1987). To mention a few, secreted EVs act primarily on target cells to transfer intercellular information via various modes of action such as internalisation, ligand-receptor interaction, secreted factors, and fusion-mediated transfer of surface receptors (Shah et al. 2019; Rezaie et al. 2022). Commonly, EVs can be classified according to their mechanism of release and size into 3 major categories; EXOs, MVs) and ABs (He et al. 2018; Hessvik and Llorente 2018). There are two distinct secretory processes concerning EXOs and MVs; EXOs are produced via the endocytic pathway and then fuse with either lysosomes or the plasma membrane (Raposo and Stoorvogel 2013; Tetta et al. 2020) in response to elevated intracellular calcium or additional downstream effects of stimuli like stress (Wei et al. 2021). MVs, on the other hand, are generated when the cell membrane protrudes outward from the cell, generating a closed sphere containing cytoplasm (Cocucci et al. 2009; Van Niel et al. 2018), and their release can be induced by various conditions, including oxidative or shear stress, hypoxia, or injury. As for ABs, they are released during apoptosis. The increased hydrostatic pressure after cell contraction causes the plasma membrane to separate from the cytoskeleton, giving rise to these entities (Doyle and Wang 2019). ABs contain materials about to be phagocytosed, such as organelles and DNA segments (Monsel et al. 2016).

### Preparation and characterisation methods of EVs

Currently, there is no universally recognised standard for isolation, characterisation, or absolute purification of EVs for large-scale clinical practice; protocols vary according to the source material, the size of sample, and the intended use of EVs (Lötvall et al. 2014; Doyle and Wang 2019). Several technologies for EVs separation are available such as ultracentrifugation, ultrafiltration, density gradient centrifugation, immunoaffinity capture, size-exclusion chromatography (SEC), and polymeric precipitation approaches. Each approach has differential advantages and drawbacks, and a combination of them may be recommended for optimum EVs enrichment (Weng et al. 2021).

#### EVs isolation methods

##### Differential centrifugation (ultracentrifugation)

Differential centrifugation is a traditional technique in term of separating EVs and it implicates utilising sequential centrifugation operations of increasing pressures to isolate EVs from impurities-containing samples with respect to their volume and physical characteristics (Revenfeld et al. 2014; De Sousa et al. 2023). This technique encompasses a preliminary phase of low-speed centrifugation to remove cell debris, followed by isolation of vesicles at 19,000–100,000 g (Xu et al. 2017). Different subsets of EVs can be enriched or concentrated, but they will not be fully isolated (Talebjedi et al. 2021). A common problem that is encountered when utilising differential ultracentrifugation is the use of large centrifugal forces which may produce clusters of vesicles as a result of rapid protein aggregation (Linares et al. 2015). Consequently, this method is better suited to laboratory settings than to clinical ones, but discrepancies might arise from several factors such as centrifugation time, speed, rotor type, and temperature, with the possibility of affecting the yield, sedimentation, and purity (Van Deun et al. 2017). In addition, this method is not suitable for isolating EVs from fluids with high viscosity, as in the case of isolating EVs from plasma (Momen-Heravi et al. 2012). Importantly, although it is still used in many studies, ultracentrifugation has been reported to significantly damage EXOs and alter their cargo (Livshts et al. 2015; Sidhom et al. 2020).

##### Density gradient centrifugation

Density gradient centrifugation (isopycnic separation and zone centrifugation) is an improved method for ultracentrifugation in which EVs are isolated according to their specific density (1.13–1.19 g/ml) in either sucrose or iodixanol solutions (Szatanek et al. 2015; Konoshenko et al. 2018). Although zone centrifugation has been demonstrated to provide greater purity and require no additional chemicals, this approach is labour-intensive, time-consuming (250 min–2 day), and suitable for high sample volumes than for clinical sample processing (De Sousa et al. 2023).

##### Ultrafiltration

Ultrafiltration, also known as microfiltration, is one of the most common approaches for isolating EVs, depending on size. It involves the use of simple membrane filters to separate EVs quickly and cheaply from larger elements in a suspension (Grant et al. 2011; Konoshenko et al. 2018). Ultrafiltration has many significant benefits: its procedures are easy to carry out, it allows for the processing of multiple samples, there are no constraints on sample volume, and the risk of EVs rupture is also considerably reduced since the vesicles are not subjected to the same pressures and pressure necessary for ultracentrifugation approach (Konoshenko et al. 2018; De Sousa et al. 2023). In spite of this, sample loss due to clogged filters and the contamination of EVS-samples with unwanted proteins are among the downsides of this approach (Carnino et al. 2019), making this technique less suitable for use in subsequent proteomic analysis if used alone (De Sousa et al. 2023).

##### SEC

SEC is a most commonly used method for isolating EVs via the fractionation or filtration of a sample through a column of porous beads, resulting in a highly pure preparation (Lozano-Ramos et al. 2015; Benedikter et al. 2017). SEC has revealed many benefits, including its high sensitivity, recoverability, reproducibility, adaptability to most laboratories, and insensitivity to highly viscous samples. SEC can also maintain vesicle integrity and contents, does not require additional chemicals, and does not cause EVs aggregation (Konoshenko et al. 2018; Varderidou-Minasian and Lorenowicz 2020; Sidhom et al. 2020; Clos-Sansalvador et al. 2022). Although SEC shows many advantages over other conventional isolation techniques, it has a few limitations such as its relatively high cost, intricate procedures, reliance on specialised apparatus and inability to discriminate between EXOs and MVs of the same size (Štulík et al. 2003; Konoshenko et al. 2018; Sidhom et al. 2020; Liangsupree et al. 2021).

##### Precipitation polymerisation

This method relies on the ability of the hydrophilic polymers such as polyethylene glycol (PEG) to diminish the solubility of the sample’s components by competing for the solvent, where it forms a mesh-like polymeric web which traps EVs in the 60–180 nm size range before pelletising and precipitating upon centrifugation at low speed (Konoshenko et al. 2018; Clos-Sansalvador et al. 2022). In order to make a two-phase method for isolation, dextran and PEG have been utilised, which has resulted in a great reduction in protein contamination (Zarovni et al. 2015). Despite its low cost, lack of specialised equipment, and the comparability between low and high sample volumes, PEG precipitation concentrates EVs, which makes it inappropriate for detecting EVs biomarker, resulting in false negatives (Coumans et al. 2017; Clos-Sansalvador et al. 2022).

##### Immunoaffinity capture (immunoaffinity purification or immunoprecipitation)

In this technique, EVs are captured by treating the sample with immunomagnetic beads that have been coated with antibodies specific to EV surface molecules (Liangsupree et al. 2021). Submicron-sized antibodies-coated magnetic beads can improve specificity, sensitivity, and yield of immunoaffinity experiments designed to isolate EVs. This method guarantees the integrity of isolated EVs regardless of vesicle size and ensures quick isolation with little effort. However, this method shows a number of limitations, including the difficulty to elute from EVs the magnetic beads and the negative impact of non-neutral pH and non-physiological salt concentrations on the biological activity of EVs (Nakai et al. 2016; Lane et al. 2017; Yoshida et al. 2017). Moreover, immunoaffinity isolation technologies remain costly, which may limit the scalability for their future clinical use (Clos-Sansalvador et al. 2022).

To date, there is no single isolation technique that can achieve high purity and yield of EVs. Therefore, coupling a good isolation method like SEC with other methods like ultracentrifugation or ultrafiltration or PEG-based retrieval can be the best solution to obtain optimal performance (Sidhom et al. 2020).

#### Purification methods of EVs

Several methods are used for EVs purification, including differential ultracentrifugation, zone ultracentrifugation, SEC, and affinity capture (Wang et al. 2021). Differential ultracentrifugation is an early, well-established, dependable method and one of the most extensively used methodologies due to its simplicity and relatively high yield (Gardiner et al. 2016). This method, however, is incapable of distinguishing particles with overlapping ranges, such as EXOs and MVs (Böing et al. 2014; Talebjedi et al. 2021; Weng et al. 2021). Zone ultracentrifugation, SEC, and filtration all face similar challenges. Unlike these previous physical separation approaches, affinity capture can separate highly pure EVs, but poor yield is obtained because of the interaction of EVs surface parameters with capture molecules linked to different carriers (e.g. magnetic beads) (Zhu et al. 2020a; Weng et al. 2021).

#### Characterisation of EVs

It is essential to perfectly characterise EVs, according to the International Society for EV`s minimal specification report, to confirm the validity of their isolation procedures and demonstrate their molecular and biological properties (Casado-Díaz et al. 2020; Weng et al. 2021). A complete EVs characterisation includes the determination of their size, shape, contents, and surface markers (Casado-Díaz et al. 2020). The general characterisation can be achieved by western blot or enzyme-linked immunosorbent assay to identify at least three positive and one negative EV protein marker, where positive protein markers should include at least one transmembrane/lipid-bound protein (e.g. CD63, CD9, CD81) and one cytosolic protein (e.g. TSG101, ALIX) (Abraham and Krasnodembskaya 2020; Weng et al. 2021). Furthermore, the single-vesicle characterisation utilises imaging techniques such as atomic force microscopy, transmission electron microscopy, and scanning electron microscopy to capture high-resolution pictures of EVs morphology. Biophysical characterisation can be also used for single-vesicle characterisation such as nanoparticle tracking analysis (NTA), dynamic light scattering, and flow cytometry (Shao et al. 2018). Although electron microscopy is currently used as the most effective way for analysing EVs’ structure, there is no single technology that could simultaneously evaluate both structural and biological features of EVs (Gurunathan et al. 2019). Other quantification and characterisation methods have been developed to analyse EVs like NTA and several optical flow-based approaches that may quantify EVs to an appropriate level, but these methods are unable to discriminate between particulate and membrane-bound vesicles, a problem which can be solved using electron microscopy (Gimona et al. 2017).

#### EVs storage and stability

Several investigations have been conducted to assess the impact of different storage temperatures (4 °C, 20 °C, and – 80 °C) and freeze–thaw cycles on the size, content, and function of isolated EVs (Jeyaram and Jay 2017). Overall, it was proved that – 80 °C is the optimal temperature for maintaining EVs’ stability and contents for downstream molecular profiling (Pinky et al. 2021; Sun et al. 2022). Freeze–thaw cycles, on the other hand, lead to the aggregation or lysis of EVs, as well as cargo loss upon their use (Kusuma et al. 2018; Gandham et al. 2020).

## Applications of MSC-EVs in different models of GI diseases

There is an increasing evidence that EVs alone are responsible for the therapeutic actions of MSCs in different GI diseases, including ALI, HF, NAFLD, and UC (Jiang et al. 2019; Du et al. 2021, 2022; Cai et al. 2021). Besides, previous studies showed that MSC-EVs can accumulate in the injured tissues and impede inflammation, apoptosis, and fibrogenesis, while modulating immune cells (Li et al. 2013; Zhao et al. 2019; Cheng et al. 2021; Shi et al. 2022). Consequently, recent researches have focussed on the use of MSC-EVs as an alternative to MSCs in the management of GI disorders (Zhao et al. 2021; Du et al. 2022; Didamoony et al. 2023).

### ALI

ALI is considered as one of the well-known life-threatening diseases that is characterised by sudden deterioration of normal liver functions, poor clinical prognosis, and high mortality (Didamoony et al. 2022). The escalation of the disease usually initiates a series of clinical syndromes, such as jaundice, coagulation disorders, hepatic encephalopathy, and ascites (Wendon et al. 2017). In ALI, multiple mechanisms work simultaneously to cause hepatic injury through inducing oxidative stress, inflammation, and apoptosis in response to infections, drugs, and chemical toxins (Basir et al. 2022; Didamoony et al. 2022). Growing evidence has indicated the successful application of MSC-EXOs in the management of ALI owing to their anti-inflammatory, anti-oxidant and anti-apoptotic features, as summarised in Table 1 (Sun et al. 2017; Zhao et al. 2019; Wu et al. 2021). Remarkably, BM-MSC-EXOs attenuated concanavalin A-induced liver injury through the improvement of tissue regeneration and the expression of anti-inflammatory cytokines and regulatory T cell (Treg) activity (Tamura et al. 2016). BM-MSC-EXOs may also reverse ALI through hindering apoptosis. The anti-apoptotic effect arises from diminishing the proapoptotic proteins B-cell lymphoma-2 (Bcl2)-associated X protein (Bax) and cleaving caspase-3, while increasing the expression of the autophagy markers, LC3-II and Beclin1, alongside with the anti-apoptotic marker; Bcl2 (Zhao et al. 2019). Interestingly, human umbilical cord MSC-EXOs was also found to exhibit desirable therapeutic effects on acetaminophen-induced ALI in vivo and in vitro via dwindling oxidative stress-induced inflammation and apoptosis after activating extracellular regulated protein kinases 1/2 and phosphoinositide 3-kinase/protein kinase B (PI3K/ AKT) trajectories (Wu et al. 2021). In this respect, human umbilical cord MSCs-EXOs enriched in glutathione peroxidase1 (GPX1) reduced oxidative stress and apoptosis in the hepatocyte and boosted protective effects of EXOs both in vivo and in vitro (Yan et al. 2017). Further studies revealed that human umbilical cord MSC-EXOs ameliorated hepatic inflammation and apoptosis in ischaemia/reperfusion (I/R) model via shuttling miR-1246 to decrease the glycogen synthase kinase 3β-mediated Wnt/β-catenin signalling (Xie et al. 2019a). Another hepatic I/R study indicated the ability of exosomal miR-1246 derived from human umbilical cord MSCs to inhibit inflammation and modulate Treg and T-helper 17 (Th17) cells balance via interleukin-6/glycoprotein130/signal transducer and activator of transcription 3 (IL-6/GP130/STAT3) axis (Xie et al. 2019b). The ability of human umbilical cord MSC-EXOs to lower aminotransferases enzymes in ALI could be also mediated via downregulating the expression of NOD-like receptor pyrin domain containing 3 (NLRP3), caspase-1, IL-1β, and IL-6 (Jiang et al. 2019). Of note, manganese superoxide dismutase in human umbilical cord MSC-EVs could dwindle the infiltration of neutrophils and mitigate apoptosis and oxidative stress (Yao et al. 2019). In case of AD-MSC-EXOs, Liu et al., (2018) found that they ameliorated lipopolysaccharide (LPS) and D-galactosamine-induced ALI in miR-17-dependent manner which reduced thioredoxin-interacting protein/ NLRP3 inflammasome activation in macrophages. More specifically, long-chain non-coding RNA H19 in AD-MSC-EVs curbed hepatic necrosis, inflammation-related cytokines, inflammatory cells infiltration and hepatocyte proliferation via hepatocyte growth factor/ hepatocyte growth factor receptor trajectory in ALI in rats (Jin et al. 2018). In term of BM-MSC-EXOs, it was documented that miR-223 prohibited NLRP3/caspase-1 signalling and suppressed inflammation-related cytokines in antigen S100-induced liver injury with consequent alleviation of hepatitis **(**Chen et al. 2018**)**.

**Table 1:** Summary of studies on the role of MSC-EVs in ALI and HF

No	Diseases	Title of study	Outcomes	References
1	ALI	Melatonin treatment enhances therapeutic effects of exosomes against acute liver ischemia–reperfusion injury	Further improvement of liver functions, anti-inflammatory, anti-apoptotic and anti-oxidants features	(Sun et al. 2017)
		Immunosuppressive effect of mesenchymal stem cell-derived exosomes on a concanavalin A-induced liver injury model	Attenuating liver injury through prohibition of apoptosis, improving of the tissue regeneration and expression of anti-inflammatory cytokines as well as increasing numbers of Treg	(Tamura et al. 2016)
		Bone marrow mesenchymal stem cell-derived exosomes attenuate D-GaIN/LPS-induced hepatocyte apoptosis by activating autophagy in vitro	Prohibition of apoptosis and ALI via energising of autophagy markers (LC3II and Beclin-1)	(Zhao et al. 2019)
		Exosomes derived from human umbilical cord mesenchymal stem cells alleviate acetaminophen-induced acute liver failure through activating ERK and IGF-1R/PI3K/AKT signaling pathway	Dwindling of oxidative stress, inflammation and apoptosis via activation of ERK1/2 and PI3K/AKT pathways, thereby attenuating the development of ALI	(Wu et al. 2021)
		HucMSC Exosome-Derived GPX1 is Required for the Recovery of Hepatic Oxidant Injury	Rescuing of the mice from ALI through curtailing of oxidative stress and apoptosis by GPX1	(Yan et al. 2017)
		Exosomes derived from human umbilical cord blood mesenchymal stem cells improve hepatic ischemia reperfusion injury via delivering miR-1246	Alleviation of hepatic IR by improving of liver function, and amending of hepatic apoptosis, necrosis and pro-inflammatory mediators via transferring exosomal miR-1246 to dwindle GSK3β-mediated Wnt/β-catenin pathway	(Xie et al. 2019a)
		Exosomal miR-1246 derived from human umbilical cord blood mesenchymal stem cells attenuates hepatic ischemia reperfusion injury by modulating T helper 17/regulatory T balance	Protection of hepatocytes from IR injury, modulating of Treg and Th17 cells’ balance to alleviate inflammation via transporting of exosomal miR-1246 targeting IL-6-gp130-STAT3 signaling, which improved the shift of Th17 towards Treg cells	(Xie et al. 2019b)
		Exosomes derived from human umbilical cord mesenchymal stem cells alleviate acute liver failure by reducing the activity of the NLRP3 inflammasome in macrophages	Repairing of the damaged hepatic tissue and reducing of the inflammation via curbing the NLRP3 inflammasome and caspase1 in vivo and in vitro	(Jiang et al. 2019)
		BMSCs-derived miR-223-containing exosomes contribute to liver protection in experimental autoimmune hepatitis	Reduction of inflammation (TNF-α, IL-17A, and IL-1β) and NLRP3/caspase-1 signalling by exosomal miR-223, thereby attenuating the development of autoimmune hepatitis	(Chen et al. 2018)
		Extracellular vesicles derived from human umbilical cord mesenchymal stem cells alleviate rat hepatic ischemia–reperfusion injury by suppressing oxidative stress and neutrophil inflammatory response	Amending of liver injury via diminishing of the neutrophils` infiltration, oxidative stress and apoptosis in hepatic tissue	(Yao et al. 2019)
		Extracellular Vesicles Secreted by Human Adipose-derived Stem Cells (hASCs) Improve Survival Rate of Rats with Acute Liver Failure by Releasing lncRNA H19	Curbing hepatic necrosis, several kinds of inflammation-related cytokines as well as chemokines and inflammatory cell infiltration beside improving of hepatocyte proliferation and anti-apoptotic effects via HGF/ c-Met trajectory	(Jin et al. 2018)
2	HF	Extracellular vesicles-derived miR-150-5p secreted by adipose-derived mesenchymal stem cells inhibits CXCL1 expression to attenuate hepatic fibrosis	Attenuating HF and curbing HSCs activation through the transfer of miR-150-5p resulting in CXCL1 underexpression	(Du et al. 2021)
		MiR-122 modification enhances the therapeutic efficacy of adipose tissue-derived mesenchymal stem cells against liver fibrosis	Improving the therapeutic efficacy of AMSCs through exosome-mediated miR-122 communication between donor AMSCs and host HSCs, dwindling of HSCs proliferation and collagen maturation, thereby lowering fibrotic alterations in the liver	(Lou et al. 2017)
		Human bone marrow mesenchymal stem cells-derived exosomes alleviate liver fibrosis through the Wnt/β-catenin pathway	Mitigation of HF by impeding collagen accumulation and HSCs activation via inhibition of Wnt/β-catenin pathway components in vivo and in vitro	(Rong et al. 2019)
		Exosomes derived from miR-181-5p-modified adipose-derived mesenchymal stem cells prevent liver fibrosis via autophagy activation	Stimulating of autophagy and reducing TGF-β1-induced HF by inhibiting the STAT3/Bcl-2/Beclin 1 pathway	(Qu et al. 2017)
		Exosomes derived from mmu_circ_0000623-modified ADSCs prevent liver fibrosis via activating autophagy	Increasing of autophagy and alleviating of HF via exosomal mmu_circ_0000623-mediated activation of miR-125/ATG4D pathway	(Zhu et al. 2020b)
		Mesenchymal stem cell-derived exosomes protect against liver fibrosis via delivering miR-148a to target KLF6/STAT3 pathway in macrophages	Suppressing pro-inflammatory macrophages, promoting anti-inflammatory macrophages and inhibiting of HF progression through miR-148a/KLF6/STAT3 pathway	(Tian et al. 2022)
		Exosomes derived from human umbilical cord mesenchymal stem cells alleviate liver fibrosis	Alleviating of HF through obstructing TGF-β1/Smad axis and epithelial-to-mesenchymal transition	(Li et al. 2013)
		HucMSC-derived exosomes delivered BECN1 induces ferroptosis of hepatic stellate cells via regulating the xCT/GPX4 axis	Mitigation of HF and stimulation of HSCs ferroptosis as well as reduction of GPX4	(Tan et al. 2022)
		HucMSC-extracellular vesicles downregulated hepatic stellate cell activation and reduced liver injury in S. japonicum-infected mice	Increasing of mice survival and enhancement of liver functions by dwindling of pro-fibrotic genes and inflammatory mediators	(Dong et al. 2020)
		MicroRNA125b-mediated Hedgehog signaling influences liver regeneration by chorionic plate-derived mesenchymal stem cells	Preventing of HF by inhibiting Smo expression and consequently hedgehog pathway activation via miR-125b	(Hyun et al. 2015)
		SEVs from tonsil-derived mesenchymal stromal cells alleviate activation of hepatic stellate cells and liver fibrosis through miR-486-5p	Preventing of HF by inhibiting Smo expression and consequently hedgehog pathway activation via miR-486-5p	(Kim et al. 2021)
		HMSCs-derived exosome circCDK13 inhibits liver fibrosis by regulating the expression of MFGE8 through miR-17-5p/KAT2B	Inhibition of HF by modulating MFGE8 expression via circCDK13/miR-17-5p/KAT2B axis	(Ma et al. 2023)

*ALI* acute liver injury, *AKT* protein kinase B, *ATG4D* autophagy related 4D cysteine peptidase, *Bcl2* B-cell lymphoma-2, *c-Met* hepatocyte growth factor receptor, *CXCL1* CXC motif chemokine-ligand 1, *ERK* extracellular regulated protein kinases, *EVs* extracellular vesicles, *GalN* galactosamine, *GPX* glutathione peroxidase, *GSK3β* glycogen synthase kinase3 beta, *hASCs* human Adipose-derived Stem Cells, *HGF* hepatocyte growth factor, *HF* hepatic fibrosis, *HSCs* hepatic stellate cells, *HucMSCs* human umbilical cord mesenchymal stem cells, *IGF-1R* insulin-like growth factor-1 receptor, *IL* interleukin, *lncRNA* human long-chain non-coding RNA, *KATB2* lysine Acetyltransferase 2B, *KLF6* Kruppel-like factor 6, *LPS* lipopolysaccharide, *MFGE8* milk fat globulin-EGF factor 8, *miR* microRNA, *NLRP3* NOD-like receptor pyrin domain containing 3, *PI3K* phosphoinositide 3-kinase, *Smo* smoothened, *STAT* signal transducer and activator of transcription, *TGF-β1* transforming growth factor-beta 1, *TH17* T-helper 17, *TNF-α* tumour necrosis factor-alpha, *Treg* regulatory T cell, *xCT* cystine/glutamate antiporter

### HF

HF is a frequent pathological condition dominated by the energization of immune cells and inflammatory-related cytokines, which leads to hepatic stellate cells (HSCs) activation and consequent extracellular matrix proteins accumulation (Acharya et al. 2021). The progression of HF results in irreversible cirrhosis, hepatocellular carcinoma (HCC), and ultimately liver failure (Doumas et al. 1971; Zhu et al. 2020b). MSC-EVs ameliorated HF in many experimental models (Table 1) by inhibiting hepatic oxidative damage, inflammatory cytokines, collagen deposition, and HSC activation as in the case of AD-MSC-EVs that curbed HSCs activation through the transfer of miR-150-5p resulting in CXC motif chemokine-ligand 1 (CXCL1) underexpression (Du et al. 2021). In addition, AD-MSC-EXOs expressing miR-122 prevented HSCs activation in HF model (Lou et al. 2017). Furthermore, Rong et al. (2019) evidenced that rat BM-MSC-EXOs can mitigate carbon tetrachloride (CCl_4_)-induced HF by impeding HSCs activation via Wnt/β-catenin pathway both in vivo and in vitro. Moreover, AD-MSC-EXOs-secreted miR-181-5p was shown to block STAT3/Bcl-2/Beclin1 pathway and increase autophagy, hence decreasing transforming growth factor-beta1 (TGF-β1)-induced HSCs activation with consequent hindrance of HF (Qu et al. 2017). In the same pattern, circular RNA mmu_circ_0000623-modified AD-MSC-EXOs was shown to activate autophagy and suppress HF by controlling miR-125 (Zhu et al. 2020b). Interestingly, MSC-EXOs derived from human umbilical cord alleviated HF through obstructing TGF-β1/Smad axis and epithelial-to-mesenchymal transition (Li et al. 2013). Specifically, miR-148a released from human umbilical cord MSC-EXOs was shown to regulate intrahepatic macrophage and control Kruppel-like factor 6/STAT3 activity and, therefore, inhibited HF progression (Tian et al. 2022). Tan et al. (2022) revealed that Beclin1 supplied by human umbilical cord MSC-EXOs led to the stimulation of HSCs ferroptosis as well as the reduction of GPX4. Furthermore, human umbilical cord MSC-EVs revealed their inhibitory effect on HF caused by Schistosoma japonicum via the downregulation of alpha-smooth muscle actin (α-SMA), collagen I, and collagen III as well as inflammatory events including interferon-gamma (IFN-γ), tumour necrosis factor-alpha (TNF-α), and IL-β1 (Dong et al. 2020). Likewise, MSC-EXOs containing miR-125b- and miR-486-5p were found to effectively prevent CCl4-induced HF by inhibiting smoothened expression and consequently hedgehog pathway activation (Hyun et al. 2015; Kim et al. 2021). In addition, Ohara et al. (2018) stated that AM-MSC-EVs improved inflammation and HF by suppressing the activation of HSCs and Kupffer cells (KCs). Recently, Ma et al. (2023) revealed that exosomal circular RNA circCDK13 from BM-MSCs inhibited HF by modulating milk fat globulin-EGF factor 8 expression via miR-17-5p/ lysine Acetyltransferase 2B axis.

### NAFLD

NAFLD is distinguished by intra-hepatocyte triglyceride buildup and concurrent immune system involvement, with consequent histological alterations, tissue destruction, and clinical symptoms due to a sedentary lifestyle and high-calorie diets (Zhao et al. 2020b; Moayedfard et al. 2022). It encompasses a cluster of disorders that range from slight steatosis (pure NAFLD) to non-alcoholic steatohepatitis (NASH), ending with cirrhosis, and HCC (Abenavoli et al. 2021; Mahmoudi et al. 2022). In the case of NASH, the development of the disease is frequently relevant to metabolic abnormalities (obesity, insulin resistance, and dysregulations of glucose and lipid metabolism). In addition, cellular and molecular changes may occur such as oxidative stress, inflammation altered immune function, and microvascular and energy dysfunction (Pouwels et al. 2022; Du et al. 2022). Generally, MSC-EVs have shown protective effects in NAFLD through controlling fat deposition-induced insulin resistance, dysregulated lipid metabolism, associated oxidative stress, and inflammatory responses, as shown in Table 2 (Niu et al. 2022; Kang et al. 2022; Du et al. 2022). Niu et al. (2022) revealed that miR-223-3p enriched in AD-MSC-EVs alleviated NAFLD by suppressing the expression of E2F transcription factor 1, hence reducing lipid buildup and HF. More importantly, human umbilical cord MSCs-derived exosomal miR-627-5p relieved liver damage in NAFLD by enhancing glucose and lipid metabolism and curbing fat mass. These metabolic outcomes emerge from the mitigation of fatty acid oxidation, mediated by the obesity-associated gene and peroxisome proliferator-activated receptor alpha (PPARα) (Cheng et al. 2021). Another study has revealed that miR-96-5p-shuttled BM-MSC-EXOs activated mitochondrial autophagy through suppressing its downstream caspase-2 (the governing player in high-fat diet-induced NASH) (El-Derany and AbdelHamid 2021). Kang et al. (2022) and Du et al. (2022) demonstrated that NASH was dramatically mitigated after using human umbilical cord MSCs-EXOs with the involvement of miR-24-3p/ Kelch-like ECH-associated protein 1(Keap1)/PPARα and nuclear factor erythroid 2-related factor 2 (Nrf2)/NADPH quinone oxidoreductase1 pathways. Besides, human umbilical cord MSC-EXOs alleviated methionine and choline-deficient diet-induced NASH in mice by upsurging the anti-inflammatory phenotype of macrophages and augmenting PPARα expression (Shi et al. 2022). Furthermore, AM-MSC-EVs significantly prevented HSCs and KCs activation and amended the degree of hepatocyte inflammation and fibrogenesis in NASH through affecting LPS/ Toll-like receptor 4 (TLR4) pathway (Ohara et al. 2018). Importantly, in a recent study, Yang et al. (2023) reported that calcium/calmodulin-dependent protein kinase 1-enriched human umbilical cord MSC-EXOs eventually prevented NAFLD in vivo and in vitro through stimulating fatty acid oxidation and inhibiting fatty acid synthesis through activation of AMP-activated protein kinase-mediated PPARα/Carnitine palmitoyltransferase 1A and sterol regulatory element-binding protein-1/fatty acid synthase pathways.

**Table 2:** Summary of studies on the role of MSC-EVs in NAFLD and UC

No	Diseases	Title of study	Outcomes	References
1	NAFLD	Adipose-derived mesenchymal stem cell-secreted extracellular vesicles alleviate non-alcoholic fatty liver disease via delivering miR-223-3p	Amelioration of NAFLD and dwindling of lipid accumulation and HF via miR-223-3p-mediated E2F1 downregulation	(Niu et al. 2022)
		Development of a non-alcoholic steatohepatitis model with rapid accumulation of fibrosis, and its treatment using mesenchymal stem cells and their small extracellular vesicles	Attenuating of NASH via reducing of inflammation and fibrosis	(Watanabe et al. 2020)
		Human umbilical cord-derived mesenchymal stem cell-exosomal miR-627-5p ameliorates non-alcoholic fatty liver disease by repressing FTO expression	Improving of glucose and lipid metabolism and alleviating of hepatic damage through miR-627-5p-mediated FTO repressing, thereby ameliorating of NAFLD progression	(Cheng et al. 2021)
		Upregulation of miR-96-5p by bone marrow mesenchymal stem cells and their exosomes alleviate non-alcoholic steatohepatitis: Emphasis on caspase-2 signaling inhibition	Upregulation of miR-96-5p that impedes caspase-2 which has a key role in inhibition of hyperlipidaemia, lowering NAFLD, hepatic apoptosis and mitochondrial mitophagy	(El-Derany and AbdelHamid 2021)
		Exosomes derived from human umbilical cord mesenchymal stem cells ameliorate experimental non-alcoholic steatohepatitis via Nrf2/NQO-1 pathway	Stimulation of Nrf2/NQO-1 pathway, thus amending of NASH-associated hepatic inflammation, lipid metabolism and oxidative stress	(Kang et al. 2022)
		Mesenchymal stem cells-derived exosomal miR-24-3p ameliorates non-alcohol fatty liver disease by targeting Keap-1	Amending of NAFLD by miR-24-3p delivery, which inhibited metabolic stress-induced oxidative stress, lipid accumulation, and inflammatory response by targeting Keap-1	(Du et al. 2022)
		Human umbilical cord mesenchymal stem cell-derived exosomes ameliorate liver steatosis by promoting fatty acid oxidation and reducing fatty acid synthesis	Preventing of NAFLD via CAMKK1-mediated lipid homoeostasis	(Yang et al. 2023)
		Extracellular Vesicles from Amnion-Derived Mesenchymal Stem Cells Ameliorate Hepatic Inflammation and Fibrosis in Rats	Mitigating of NASH by reducing of inflammation and fibrosis	(Ohara et al. 2018)
		Human umbilical cord mesenchymal stromal cell-derived exosomes protect against MCD-induced NASH in a mouse model	Alleviating of NASH by augmenting of the anti-inflammatory phenotype of macrophages and upregulating of PPARα protein expression	(Shi et al. 2022)
2	UC	Exosomes Derived from Human Umbilical Cord Mesenchymal Stem Cells Relieve Inflammatory Bowel Disease in Mice	Mitigation of DSS-induced IBD via suppressing of IL-7 expression in macrophages which alleviate inflammatory responses	(Mao et al. 2017)
		Exosomes derived from human umbilical cord mesenchymal stem cells alleviate inflammatory bowel disease in mice through ubiquitination	Repairing damaged tissue in the colon and spleen, ameliorating of DSS-induced IBD via inhibition of ubiquitination	(Wu et al. 2018)
		HucMSC-exosomes carrying miR-326 inhibit neddylation to relieve inflammatory bowel disease in mice	Inhibition of neddylation process-induced NF-ĸB activation and relieving DSS-induced IBD via miR-326	(Wang et al. 2020)
		hucMSC-derived exosomes attenuate colitis by regulating macrophage pyroptosis via the miR-378a-5p/NLRP3 axis	Improving IBD by inhibiting macrophage pyroptosis via the miR-378a-5p/NLRP3 axis	(Cai et al. 2021)
		A novel therapeutic approach for inflammatory bowel disease by exosomes derived from human umbilical cord mesenchymal stem cells to repair intestinal barrier via TSG-6	Protecting against IBD through restoring intestinal mucosal barrier function and homeostasis of the intestinal immune system via TSG-6	(Yang et al. 2021)
		Olfactory Ecto-Mesenchymal Stem Cell-Derived Exosomes Ameliorate Experimental Colitis via Modulating Th1/Th17 and Treg Cell Responses	Inhibition of T-cells proliferation and differentiation, endorsing of activated T-cells apoptosis as well as inducing of Tregs cells and eventually alleviating of colitis and promoting the repair of damaged intestinal tissues	(Tian et al. 2020)
		Extracellular vesicles containing miR-146a attenuate experimental colitis by targeting TRAF6 and IRAK1	Assuaging of colitis by targeting TRAF6 and IRAK1 expression via miR-146a	(Wu et al. 2019)
		Extracellular vesicles derived from bone marrow mesenchymal stem cells attenuate dextran sodium sulfate-induced ulcerative colitis by promoting M2 macrophage polarization	Diminishing of the inflammation in DSS-induced UC by endorsing M2 macrophage polarization via modulating the JAK1/STAT1/STAT6 axis	(Cao et al. 2019)
		Adipose-derived mesenchymal stem cell-secreted exosome alleviates dextran sulfate sodium-induced acute colitis by Treg cell induction and inflammatory cytokine reduction	Reduction of Th17 production, and arousing the Treg cells percentage, and thus ameliorating acute colitis	(Heidari et al. 2021)
		Human Adipose Mesenchymal Stem Cell-derived Exosomes Protect Mice from DSS-Induced Inflammatory Bowel Disease by Promoting Intestinal-stem-cell and Epithelial Regeneration	Homing to the inflammatory sites of the colorectal tissue, inhibiting inflammatory cell infiltration and colonic inflammation, preventing alterations of colon length and crypt loss, preventing rectal bleeding and reducing histological scores of DAI	(Yu et al. 2021)

*CAMKK1* calcium/calmodulin-dependent protein kinase 1, *DAI* Disease Activity Index, *DSS* dextran sulfate sodium, *FTO* fat mass and obesity-associated gene, *HF* hepatic fibrosis, *HucMSC* human umbilical cord mesenchymal stem cell, *IBD* irritable bowel disease, *IL-7* interleukin-7, *IRAK1* IL-1 receptor-associated kinase 1, *JAK*1 Janus kinase 1, *Keap1* Kelch-like ECH-associated protein 1, *NAFLD* non-alcoholic fatty liver disease, *NASH* non-alcoholic steatohepatitis, *NF-ĸB* nuclear factor-kappa B, *NLRP3* NOD-like receptor family, pyrin domain containing 3, *Nrf2* nuclear factor erythroid 2-related factor 2, *NQO-1* NADPH quinone oxidoreductase 1, *MCD* methionine–choline-deficient diet, *miR* microRNA, *PPARα* Peroxisomal proliferator-activated receptor alpha, *STAT* signal transducer and activator of transcription, *Th1* Type 1 T-helper, *Th17* T-helper 17, *TRAF6* TNF receptor-associated factor 6, *Treg* Regulatory T cells, *TSG-6* tumour necrosis factor-α stimulated gene 6, *UC* ulcerative colitis

### Ulcerative colitis (UC)

UC is one of the common forms of IBD which is manifested by recurrent inflammation and ulceration of the colonic mucosa with varying extension from the rectum towards the cecum (Owusu et al. 2020; Guo et al. 2022). Untreated UC may give rise to increased risk of developing colorectal cancer (Olén et al. 2020). Inflammation and oxidative stress are vital factors in the pathogenesis of UC (Soubh et al. 2015; Arafa et al. 2020; De Oliveira et al. 2021) and are considered the key targets of MSC-EVs therapy (Yang et al. 2015; Xia et al. 2021; Zhu et al. 2022). The therapeutic efficacy of MSC-EVs was related to EVs autonomous targeting capabilities to reach the injured colon tissues, reduce inflammatory cell infiltration, and, hence, maintain the integrity of colonic mucosa and mitigate the severity of UC symptoms (Table 2) (Yang et al. 2015; Heidari et al. 2021; Cai et al. 2021). For instance, Mao et al. (2017) reported the amelioration of dextran sulfate sodium (DSS)-induced UC by human umbilical cord MSC-EXOs through profound decline in the recruitment of inflammatory M1 macrophages to the damaged colon and diminished pro-inflammatory cytokines release such as TNF-α, IL-1*β*, and IL-6. Similarly, the study of Wu et al. (2018) stated that human umbilical cord MSC-EXOs amended UC and inhibited ubiquitin-associated molecules (K48, K63, and FK2)-mediated inflammation through turning off nuclear factor-kappa B (NF-κB) and mammalian target of rapamycin trajectories. Human umbilical cord MSC-EXOs enriched in miR-326 also relieved DSS-induced IBD in mice by preventing the binding of NEDD8 to cullin 1 (neddylation process) as well as NF‐κB signalling (Wang et al. 2020). Noteworthy, human umbilical cord MSC-EXOs mitigated DSS-induced IBD by lessening macrophage pyroptosis via modulation of miR-378a-5p/NLRP3 pathway (Cai et al. 2021). Moreover, Yang et al. (2021) pointed to the anti-inflammatory effect of TNF-α-stimulated gene 6 in human umbilical cord MSC-EXOs that succeeded to mitigate UC in addition to intestinal function and immune homeostasis. In addition, olfactory ecto-MSCs-EXOs alleviated experimental colitis via suppressing differentiation of proinflammatory Th1/Th17 cells and inducing differentiation of anti-inflammatory Treg cells (Tian et al. 2020). Furthermore, BM-MSCs-EXOs were reported to alleviate DSS-induced UC by endorsing M2 macrophage polarisation and modulating Janus kinase 1/STAT1/STAT6 axis (Cao et al. 2019).

In summary, researches that compared EVs to their parent cells demonstrated a more pronounced or a comparable efficacy for the derived EVs with a better safety profile. Obviously, Fattore et al. (2015) verified that MSC-EVs reveal more immunomodulatory effects compared to their parent cells through enhancement of CD4^+^, CD25^+^ and CD127^low^ Tregs and anti-inflammatory cytokines. As well, another study proved that MSC-EVs surpassed the lung vasculature and improved HF and restored its function with comparable efficacy to their parent MSCs through targeting various cell types in liver (Rostom et al. 2020; Gupta et al. 2022). Notably, the absence of major histocompatibility complex class I-II on allogeneic and autologous MSC-EVs makes them more safely applied as evidenced by Sengupta et al. (2020) using allogeneic BM-MSC-EVs in COVID-19 patients in a prospective non-randomised cohort study with no demonstrated adverse effects and satisfied safety endpoints. On the other hand, MSCs show many safety concerns like their potential for aberrant differentiation or spontaneous malignancy which have encouraged the replacement of MSCs by EVs, although some clinical results still support the safety of MSCs application (Karussis et al. 2010; Lalu et al. 2012; Kim et al. 2017; Hosseini et al. 2022). Maji et al. (2017) and Sun et al. (2016) also revealed no genotoxic effects or detrimental effects of MSC-EVs on liver and kidneys both in vitro and in vivo, respectively.

## Strategies to enhance the therapeutic potential of MSC-EVs

Utilising MSC-EVs in various diseases is limited by accumulating drawbacks that restricted their large-scale applications. These drawbacks include the low yield of MSC-EVs under conventional culture media (Madrigal et al. 2014) and the marked decrease in the therapeutic effect of secreted EVs from MSCs senescence following multiple generations of cultures in vitro (Joo et al. 2020). In addition, the poor targeting characteristics to the site of injury after i.v. administration is regarded as inherent properties of native or unmodified EXOs (Borrelli et al. 2018; Xu et al. 2020). Besides, the diminished efficacy of EVs may arise from their degradation in response to increased oxidative stress under pathological conditions and its consequent cellular autophagy activation (Zhang et al. 2022). Accordingly, it is highly recommended to broaden the clinical applications of MSC-EVs and improve their therapeutic efficacy (Lopez-Santalla and Garin 2021; Didamoony et al. 2023).

### Preconditioning approaches of MSC-EVs

**Fig. 1:**
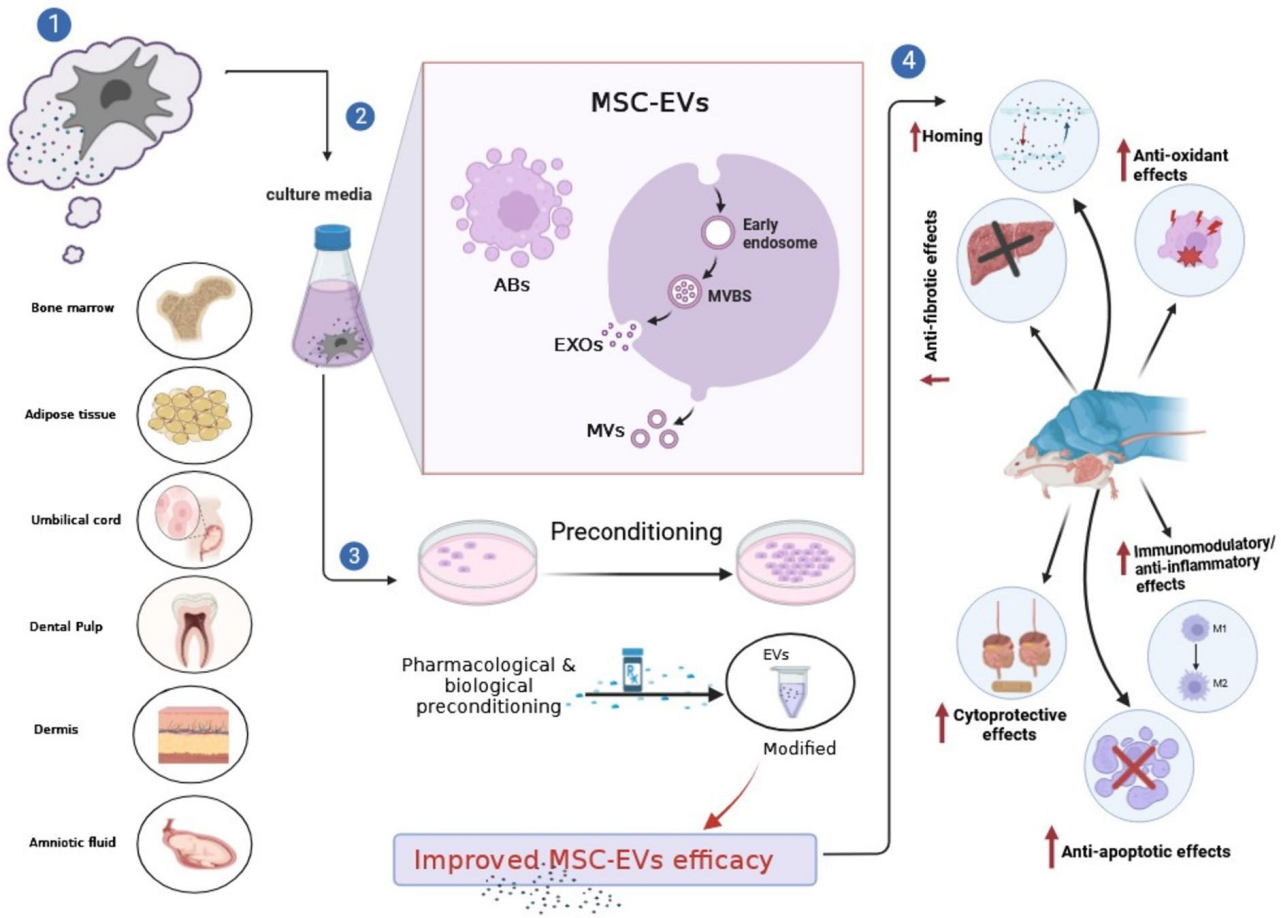
Sources of MSCs and their derived EVs as well as mechanisms of enhancement of their functions using preconditioning approaches. *ABs* apoptotic bodies, *EXOs* exosomes, *MSC-EVs* mesenchymal stem cell-derived extracellular vesicles, *MVBs* multivesicular bodies, *MVs* microvesicles

Preconditioning is a process encompassing enhancement of the therapeutic efficacy and regenerative abilities of the administered stem cells or their derivatives and can be accomplished by two cytoprotective strategies; the first involves augmenting particular valuable cell trajectory, and the second one is achieved by providing sublethal environment to adapt cells to harsh environment to which they are subjected during pathological conditions (Tilkorn et al. 2012; Touani et al. 2021; Moeinabadi-Bidgoli et al. 2021). Since the characteristics of MSC-EVs are mainly dependent on MSCs status, the preconditioning of MSCs with chemical agents, biomolecules, or cytokines could improve the immunomodulatory activities as well as the reparative and regenerative effects of their derived EVs, a part of MSCs paracrine system (Fig. 1) (Noronha Nc et al. 2019). Importantly, pharmacological preconditioning appears to be a reasonably affordable and a valuable technique that can be applied clinically without the use of sophisticated protocols or specific instrumentations (Chen et al. 2022b).

#### Pharmacological preconditioning

Preconditioning of MSCs in vitro with drugs or natural medications was documented to enhance the MSCs-EVs therapeutic effects as shown in multiple diseases by modifying various pathways and restoring the lost functions (Hu and Li 2018; Harrell et al. 2019a, b). Optimising MSC-EVs composition is one of the important outcomes of in vitro MSCs preconditioning that result in developing disease-specific, MSC-based, and cell-free products (Harrell et al. 2019a, b). For example, the natural yellow agent obtained from the spice turmeric, curcumin (Cur), provided EXOs with superior effects for NASH treatment using Cur-pre-treated MSCs via amendments of hepatic fibrogenesis, inflammation, oxidative stress in vivo (Motterlini et al. 2000). In addition, Cur-EXOs repressed lipid synthesis genes such as PPAR-α and inverted the lipotoxic effect of palmitic acid-treated HepG2 cells and mitochondrial-dependent apoptosis in vitro, as compared to native MSC-EXOs (Tawfeek and Kasem 2023). In the same manner, the preconditioning of MSCs with baicalin, a flavonoid isolated from roots of *Scutellaria baicalensis,* produced a remarkable enhancement in the function of their derived EXOs in comparison with unmodified EXOs. This was justified by improving liver functions in ALI through activating p62/Keap1/Nrf2 signalling and inhibiting oxidative burst, inflammation, and lipid peroxidation-induced ferroptosis (Zhao et al. 2022).

Preconditioning with pharmacological agents in vivo robustly urges the survival and therapeutic efficacy of MSCs and their derivatives (Mortezaee et al. 2017; Feng et al. 2018; Yousefi-Ahmadipour et al. 2019). This was evident by using rupatadine, an antihistaminic drug which enhanced the therapeutic effects of MSC-EXOs in vivo against HF in rats as compared to conventional MSC-EXOs. Rupatadine provided a more favourable environment by elevating miR-200a level and hampering oxidative stress, inflammation (platelet activating factor/TNF-α), necroptosis (receptor-interacting protein kinase 3/mixed lineage kinase domain-like protein), and hedgehog pathway with consequent anti-fibrogenic action (Didamoony et al. 2023). Similarly, Wei et al. (2020) demonstrated that combining MSC-EXOs with glycyrrhetinic acid (a triterpenoid saponin isolated from the root and rhizome extracts of liquorice) significantly reinforced the expression of proteins with anti-inflammatory activities and restored the expression of dysregulated proteins associated with inflammation and oxidative stress, resulting in further improvement of MSC-EXOs therapeutic potential in liver injury both in vivo and in vitro. Moreover, utilising nilotinib, a second-generation tyrosine kinase inhibitor, with MSC-EXOs therapy improved the anti-fibrotic effect of EXOs in CCl4-induced HF through inhibiting oxidative stress, inflammation, and apoptosis in comparison with MSC-EXOs therapy alone (Shiha et al. 2020). Furthermore, Chang et al. (2019) proved that combining MSC-EXOs with melatonin, a mitochondrial hormone secreted by the pineal gland (Lopez-Santalla and Garin 2021), alleviated the inflammatory status, apoptosis, and colon injury in rats subjected to DSS, an effect that was better than that obtained using unmodified EXOs. Besides, combining green tea with MSC-EXOs produced better EXOs tolerance to lethal oxidative stress and inflammation (CXC receptor 2 and TLR4), and hence, more pronounced therapeutic potential against UC in rats (El-Desoky Mohamady et al. 2022).

#### Preconditioning with other mediators

Improving the paracrine efficiency of MSCs results in a consequent enhancement of their derived EXOs therapeutic activity which can be attained by the aid of biological molecules or mediators being one of the preconditioning strategies. Hydrogen sulphide is one of the metabolites produced by the cells during pathological conditions such as ischaemia and oxidative stress. Surprisingly, this mediator possesses ROS scavenging role leading to enhanced cell resistance against hypoxia and oxidative stress (Zhang et al. 2016; Scammahorn et al. 2021). Accordingly, transplantation of the derived EXOs resulted from preconditioning of MSCs with sodium hydrosulfide revealed superior hepatoprotective and immunosuppressive effects as compared to unmodified EXOs via upregulation of the expression of long non-coding RNA metastasis-associated lung adenocarcinoma transcript 1 and anti-apoptotic factor Bcl2 in addition to downregulation of the expression of apoptotic proteins (cleaved caspase-3, Bax and Bcl-2 homologous antagonist/killer1) (Sameri et al. 2022). Growth factors, a vital group of biological mediators, were also found to modulate signal transduction involved in cell growth, proliferation, survival, and other regenerative-related capacities (Hu and Li 2018). In comparison with unmodified MSCs-EXOs, preconditioning of Wharton’s jelly-MSCs with TGF-β1 produced EXOs with maximum repressive effect on TGF-β1/Smad3 axis and fibrotic markers (α-SMA, type I collagen-alpha 1, E-cadherin) in activated LX-2 cells (Bavarsad et al. 2022).

Furthermore, cytokines such as TNF-α, IL-6 and IFN-γ are mediators that improve the regenerative capacity and therapeutic potential of MSC-EXOs. This was observed utilising MSC-EXOs preconditioned with IFN-γ in a murine model with liver cirrhosis which revealed alleviation of both inflammation and fibrosis (Takeuchi et al. 2021). Likewise, EXOs derived from TNF-α-treated MSCs afforded improved therapeutic potential in a mouse model of ALI as compared to untreated EXOs. These outcomes were related to more pronounced overexpression of miR-299-3p which in turn inhibited the recruitment and activation of NLRP3-related inflammatory pathway (Zhang et al. 2020). Similarly, Shao et al. (2020) demonstrated experimentally the ability of IL-6 pre-treated human umbilical cord MSC-EXOs to diminish the generation of inflammatory cytokines via miR-455-3p which targeted the IL-6-related signalling cascades in ALI. In addition, LPS-preconditioned MSC-EXOs mitigated inflammation and the severity of UC compared to ordinary/unmodified MSC-EXOs (Gu et al. 2021). Another study reported that IFN-γ enhanced the therapeutic efficacy of MSC-EXOs for management of colitis in mice through overexpressing miR-125a and miR-125b in MSC-EXOs which directly acted on STAT3 and repressed Th17 cell differentiation as well as inflammation (Yang et al. 2020).

## Current challenges of clinical applications of MSC-EVs

MSC-EVs are characterised by similar or even better function in comparison with their parent cells because of their higher biocompatibility, greater trajectory in intercellular communication, and higher efficiency in drug delivery (Cheng et al. 2020; Racchetti and Meldolesi 2021; Yin et al. 2023). Furthermore, MSC-EVs showed no evidence of spontaneous oncogenic potential or any negative immune responses (Cheng et al. 2020; Hou et al. 2021; Yin et al. 2023). On the other hand, MSCs can promote and aggravate tumour growth as demonstrated experimentally in several types of cancer such as breast and colorectal cancer in addition to gastric carcinoma (Karnoub et al. 2007; Quante et al. 2011; De Boeck et al. 2013; Musiał-Wysocka et al. 2019). More importantly, MSC-EVs are easy to store with extreme stability and without using harmful cryopreservatives (Cheng et al. 2020). MSC-EVs also exhibit good penetration of biological barriers and revealed minimal risk of microvascular embolism as compared to their parent MSCs which caused instant blood-mediated inflammatory reaction upon intravenous administration in different experimental studies (Fiedler et al. 2018; Musiał-Wysocka et al. 2019; Han et al. 2020; Sun et al. 2022), leading to pulmonary embolism (Tatsumi et al. 2013). Thus, MSC-EVs show a superior safety profile making them a promising therapeutic approach for a wide range of diseases or disorders (Zhu et al. 2017; Psaraki et al. 2022).

Despite all these advantages, there are numerous challenges that should be overcome before the clinical application of MSC-EVs in GI diseases. These concerns stem from: (a) the inability to choose the optimal EVs source due to the lack of clear comparison among different MSCs sources (Bruno et al. 2020); (b) the molecular heterogeneity in EVs preparations because of the difference in methods of EVs isolation, purification, and characterisation—which contradicts with the homogeneity required for clinical practice (Abraham and Krasnodembskaya 2020; Guo et al. 2021); (c) the difficulty to ascertain the optimal route of delivery and the therapeutic dosage required for each GI condition, which remains a mystery to clinicians due to the lack of well-recognised and standardised techniques for EVs isolation and characterisation (Guo et al. 2021; Ahmed and Al-Massri 2022); (d) the contamination of EVs preparations with apoptotic cells fragments, lipoproteins, or proteins (Choi et al. 2015; Hou et al. 2021); (e) a dearth of techniques for large-scale EVs production and extraction (Guo et al. 2021; Williams et al. 2023); (f) a scarcity of information about the exact content within MSC-EVs which can vary greatly due to different sources and conditions (Cheng et al. 2020); (g) the low temperatures during handling and transplantation, and the freeze–thaw cycles which can induce EVs clumping and cargo degradation (Pinky et al. 2021).

Therefore, from a practical standpoint, the apparent insignificant results of MSC-EVs in clinical trials could be related to the disease stage, the timing of their injection, the dose used, and the source of the MSC-EVs either from healthy or diseased cells. Further investigations are needed to scale up and optimise specific and standardised methodologies for MSC-EVs production, isolation, purification, and characterisation. Besides, it is important to validate the dosage and half-life of MSC-EVs and evaluate alternative approaches for EVs storage to enhance their stability. It is also necessary to examine the potential impacts of EVs derived from different sources of MSCs in various GI disorders and to investigate new techniques for modulating MSC-EVs composition and their biological activity. Furthermore, specific and effective markers for analysing EVs at a single-vesicle level should be identified to distinguish EVs source, ensure their purity, and preclude unknown harmful impacts of their use.

## Conclusion

Because of the alarming rise in incidence and prevalence of GI diseases, researchers have been working to identify new approaches for the management of these diseases. MSC-EVs represent an attractive therapeutic paradigm for treating various GI diseases through maintaining the therapeutic advantages of their parents MSCs, but with reduced risks of iatrogenic tumour formation, immunogenicity, and microvascular obstructions. MSC-EVs restore homeostasis and enable the injured cells to recover through their anti-oxidant, anti-apoptotic, anti-inflammatory, anti-fibrotic, and immunomodulatory actions. Besides, the therapeutic efficacy of MSC-EVs can be improved by the preconditioning approach which utilises pharmacological agents or biological mediators to adapt them to the lethal environment to which they are subjected during pathological conditions. Notably, there have been tremendous efforts to improve the separation and production yield of MSC-EVs as well as their efficacy and stability over time following in vivo transplantation. Despite all these efforts, additional studies and methodologies are still needed to overcome the challenges and difficulties of their clinical applications.

## Data Availability

All data are available in the manuscript.
